# Supplementary material for: Prevalence of Locomotive Organ Impairment and Associated Factors among Middle-Aged and Older People in Nan Province, Thailand
Source: Int J Environ Res Public Health. 2021 Oct 15;18(20):10871. doi: 10.3390/ijerph182010871 (PMC8536190; doi:10.3390/ijerph182010871)
Supplement: Supplementary file 1 [file ijerph-18-10871-s001.zip › ijerph-1371416-supplementary.pdf]

International Journal of
Environmental Research and Public Health

MDPI

Supplemental Online Table S1. Socio-demographic characteristics of the participants (n = 165)			
	**Variables**	***n***	**%**
**Age**	50–59	40	24.2
Mean ± SD: 65.2 ± 8.1	60–69	68	41.2
	70–79	49	29.7
	≥80	3	1.8
**Sex**	Male	51	30.9
	Female	114	69.1
**Marital status**	Single	14	8.5
	Married	112	67.9
	Widowed	33	20.0
	Divorced (separated)	6	3.6
**Number of Family members**	1	7	4.2
Mean ± SD: 3.4 ± 1.6	2	51	30.9
	3–5	92	55.8
	≥6	15	9.1
**Educational Attainment**	Primary school completed	99	60.0
	Secondary school completed	32	16.4
**Years of schooling**	Vocational completed	6	3.6
Median 6.7（IQR: 4.0–9.0）	University, bachelor's degree completed	19	11.5
	University and above completed	1	0.6
	No school	8	4.9
**Economic status**	Financially difficult	10	6.2
(n = 161)	Slightly difficult	55	34.2
	Slightly comfortable	46	28.9
	Financially comfortable	50	31.1
**Current job**	Agriculture	41	24.9
	Labour	28	17.0
	Merchant	21	12.7
	Housewife	8	4.9
	Desk work	0	0.0
	Other	4	2.4
	Unemployed	63	38.2
**Comorbidities**	Hypertension	89	54.6
(n = 163)	Hyperlipidaemia	60	36.8
	Diabetes	34	20.9
	Cardiovascular disease	5	3.1

	Stroke	4	2.5
	Chronic obstructive pulmonary disease	2	1.2
	Cancer	2	1.2
	Other	17	10.4
	No underlying disease	51	31.3
**BMI (kg/m^2)**	<18.5	6	3.6
Mean ± SD: 24.0 ± 3.4	18.5–24.9	100	60.6
	≥25.0	59	35.8

SD, standard deviation; IQR, interquartile range; BMI, body mass index
